# Supplementary material for: Mechanical forces drive ordered patterning of hair cells in the mammalian inner ear
Source: Nat Commun. 2020 Oct 12;11:5137. doi: 10.1038/s41467-020-18894-8 (PMC7550578; doi:10.1038/s41467-020-18894-8)
Supplement: Supplementary file 1 — Supplementary Information [file 41467_2020_18894_MOESM1_ESM.pdf]

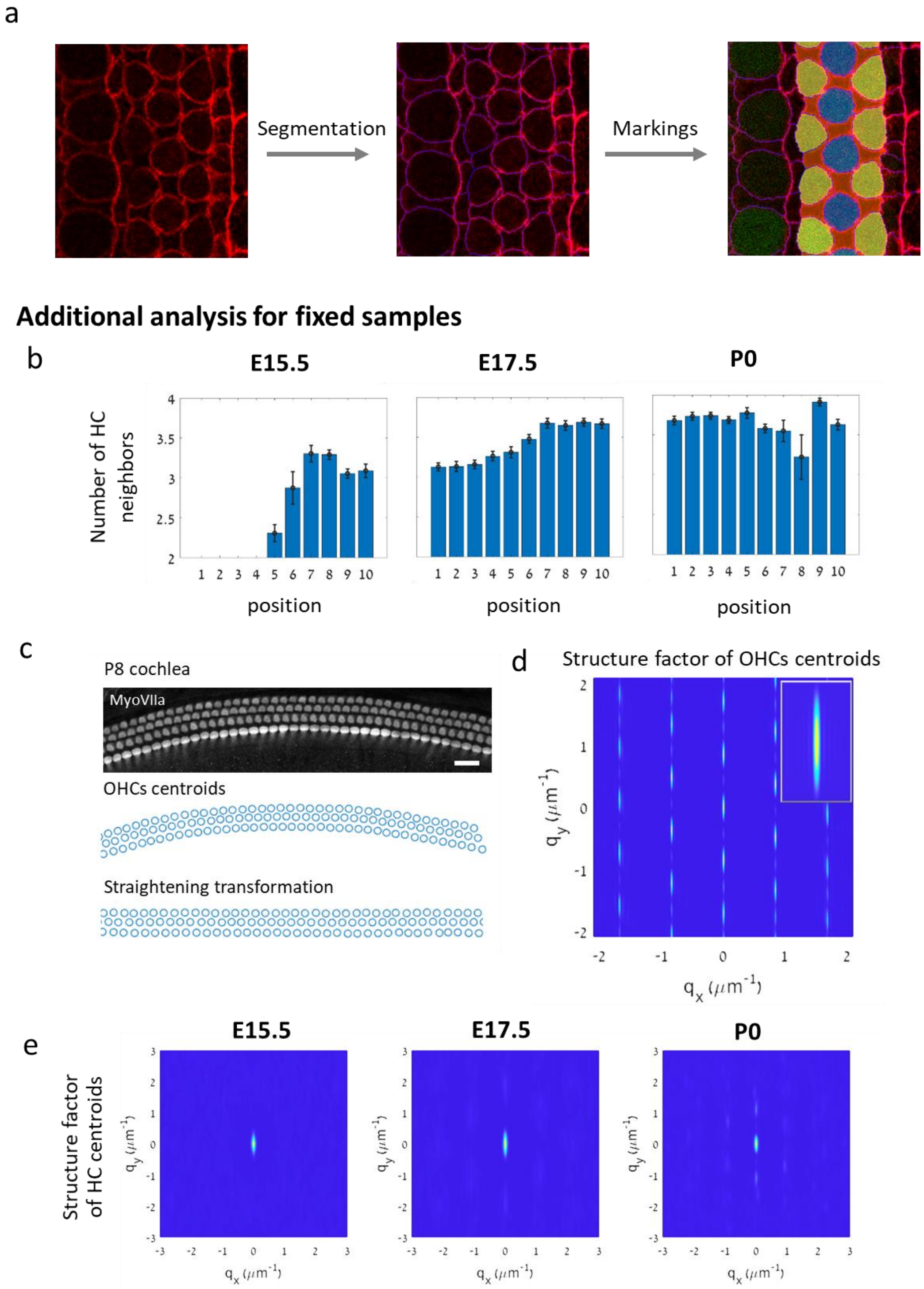

Figure S1. Description of image analysis sequence and additional morphological data on HCs and SCs. (a) Schematic of the segmentation process. The borders of cells are delineated (purple lines) and different cell types are marked (yellow/orange/blue cells) using a semi-automatic code. **(b)** Number of HC neighbors of SCs from the OHC_2 row in different regions of the cochlea from apex to base at E15.5, E17.5, P0. Local measures of number of HC neighbors associated with each SC are pooled by developmental age over n=3,4,3 cochleae at E15.5, E17.5, P0, respectively, and then binned by cochlear position. Bars represent average on all local measurements within each bin. Error bars represent S.E.M. This

analysis shows that as development progresses the number of HC neighbors of SCs increases. **(c)** An image of a mid region from a fully developed cochlea at P8, stained with MyoVIIa (marker for HCs) and taken at the apical side of the tissue (top). Scatter plots show the centroids of the OHCs apical cross section before (middle) and after (bottom) applying straightening transformation (see methods). Scale bar: $20\mu m$. **(d)** Structure factor of the transformed OHCs centroids in (b). The observed Bragg peaks demonstrates the high level of crystalline order in the system. Image in inset shows a closeup of the center peak with its adjacent sub peaks, as expected from a finite size system. **(e)** Structure factor of OHCs from the base-mid region at E15.5, E17.5, P0. The emergence of peaks in the structure factor shows the gradual formation of a crystal-like structure of OHCs. The data is summed over n=3,4,3 cochleae at E15.5, E17.5, P0, respectively.

Additional apical area analysis

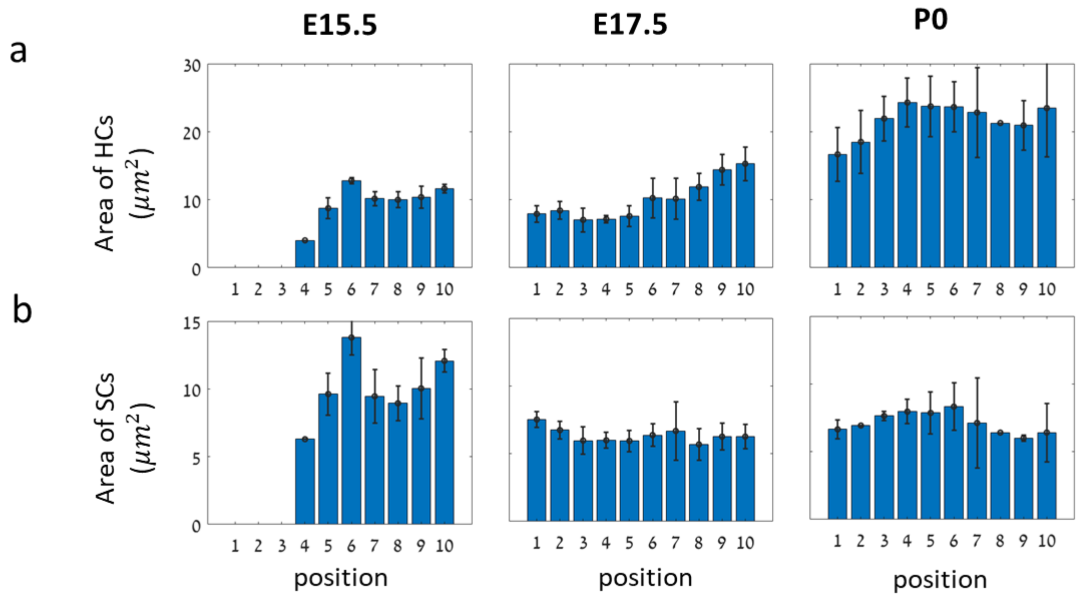

Figure S2. Analysis of changes is apical surface area. (a-b) Apical surface area of HCs (a) and SCs (b) in different regions of the cochlea from apex to base at E15.5, E17.5, P0. This analysis shows that as development progresses, HC apical surface area increases while SC apical surface area slightly decreases. Local measures of apical surface area associated with each HC or SC are pooled by developmental age over n=3,4,3 cochleae at E15.5, E17.5, P0, respectively, and then binned by cochlear position. Bars represent average on all local measurements within each bin. Error bars represent S.E.M.

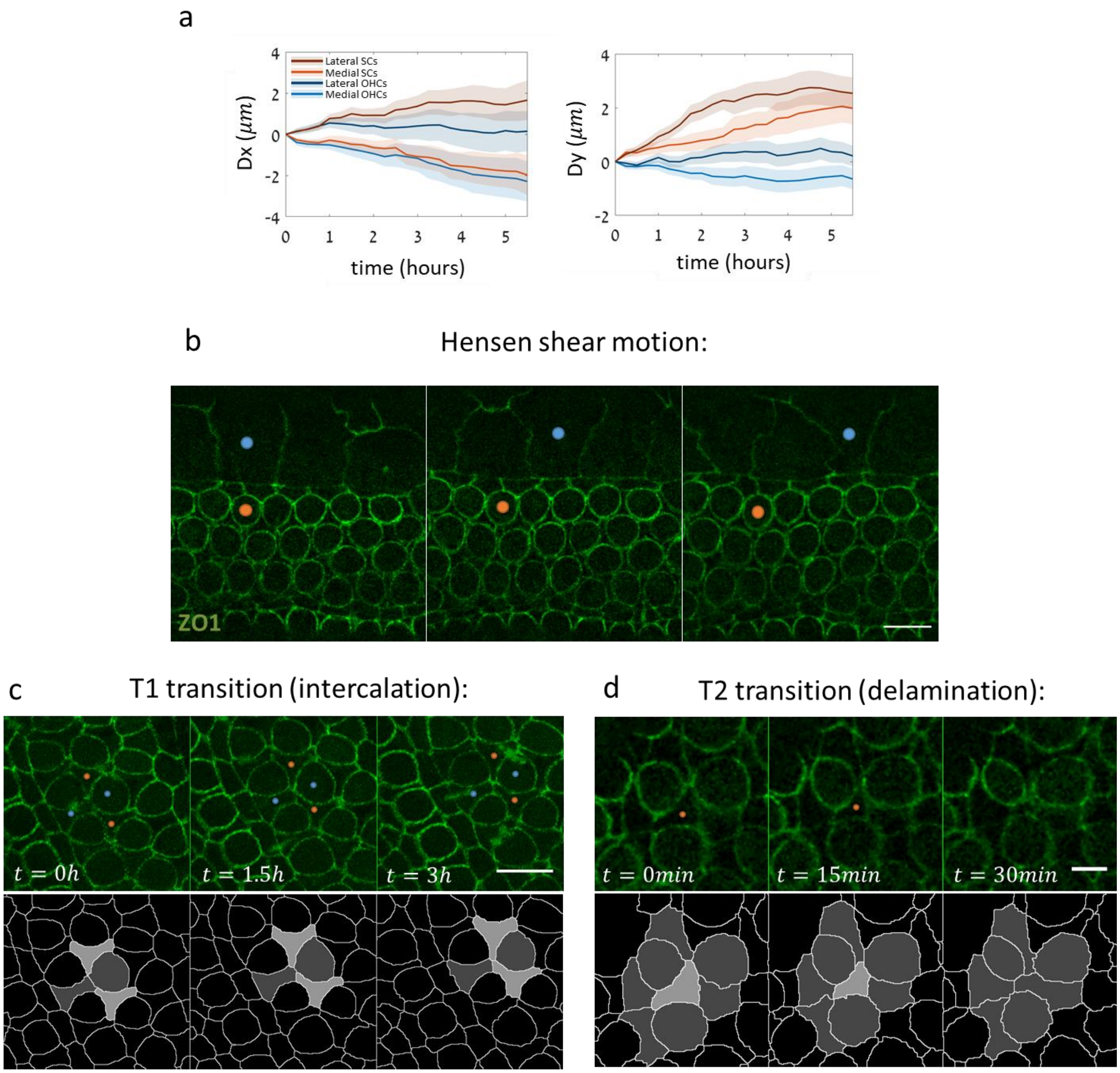

Figure S3. Additional data on shear movement, intercalation and delaminations. (a) Displacement of HCs and SCs acquired from another time-lapse movie of an E15.5 cochlear explant (different sample than Fig. 2a-b). Displacements are calculated relative to the initial position of each cell. Similar to the sample analyzed in Fig. 2, cells from the medial and lateral OHC regions exhibit different behaviors. Shaded region around every line represents the boundaries of S.E.M. **(b)** Filmstrip showing sliding motion of Hensen cells on E17.5 cochlear explant. Blue and red dots mark initially aligned Hensen cell and OHC, respectively. Movie shown in Supplementary Video 2. Scale bar: $10\mu m$. **(c)** A filmstrip from a E17.5 mouse cochlear explant showing an intercalation process (T1 transition) between the cell pair marked in red dots and the cell pair marked in blue dots. Bottom row shows a segmented version of the intercalation process. Movie shown in Supplementary Video 5. Scale bar: $10\mu m$. **(d)** A filmstrip from a E17.5 mouse cochlear explant showing a delamination process (T2 transition), where the cell marked with red dot decreases in surface area and eventually vanishes from the apical surface. Bottom row presents a segmented version of the transition. Movie shown in Supplementary Video 6. Scale bar: $5\mu m$.

a

2D vertex model:

Dynamics is determined by minimizing the system's energy under external forces:

$$E = \sum_{n=1}^{N_c}\left[\frac{1}{2}\alpha_n\left(A_n - A_{n,0}\right)^2 + \sum_{\langle ij\rangle_n}\gamma_n^{ij} l_n^{ij} + \frac{1}{2}\Gamma_n L_n^2 + \sum_{m=1}^{N_c}\sigma_{nm}\left(\frac{D_{nm}}{R_{nm}}\right)^{\kappa}\right]$$

$$\left[\vec{F}_n^{ext}\right]_i = \eta_n y_n^{CM}\hat{x} + \zeta_n y_n^{CM}\vec{\nabla}_i y_n^{CM}$$

For each vertex:

$$-\vec{\nabla}E + \vec{F}^{ext} = 0$$

Where n, m are cell indices, N_c is the total number of cells and $\langle ij\rangle_n$ are the pairs of adjacent vertices in cell n.

Variables (determined by vertices):

A_n - area of cell n
l_n^{ij} - length of junction ij
L_n - perimeter of cell n
y_n^{CM} - y coordinate of center of mass (relative to PCs)
R_{nm} - distance between cells n and m

Parameters:

α_n - incompressibility
$A_{n,0}$ - preferable area
γ_n^{ij} - tension of bond ij
Γ_n - structural rigidity
η_n - shear force
ζ_n - compression coefficient
σ_{nm} - repulsion coefficient
κ- repulsion coefficient
D_{nm} - repulsion distance

b

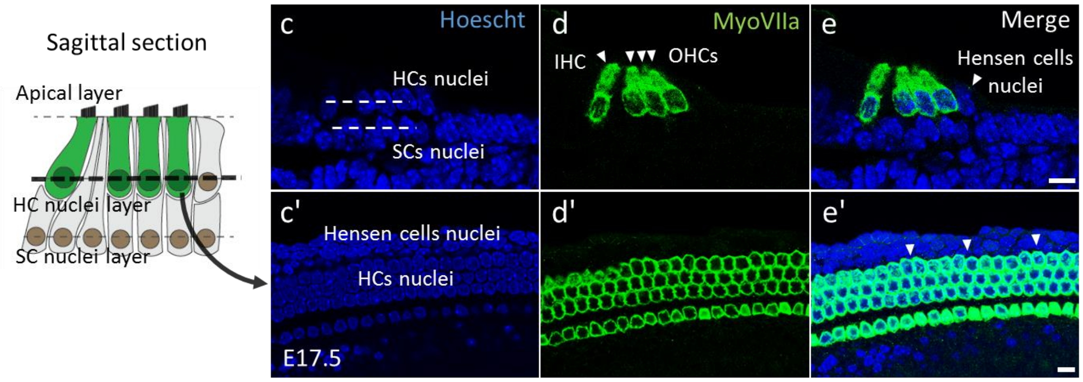

Figure S4. Mathematical description of the model and 3D geometry of the organ of Corti. (a) The dynamics of the vertices (e.g. their position) is determined by minimizing the mechanical energy function of the system under external forces. The energy function includes terms corresponding to internal pressure (depends on cell area), junction tension (depends on junction length), cell contractility (depends on cell perimeter), and repulsion forces between HCs (depend on distance between HCS). In addition to the energy function there are external forces (non-conserved) that correspond to horizontal shear and vertical compression (both depend on distance from pillar cells). Bottom: list of variables (depend on vertex positions) and parameters of the model. **(b)** Schematic of a sagittal section of the organ of Corti showing the apical layer, HC nuclei layer and SC nuclei layer. **(c-e')** Images of E17.5 cochleae at the mid region showing a sagittal section of the organ of Corti (c-e) and a whole mount confocal image taken at the HC nuclei plane (c'-e'). The cochleae were stained with Hoescht (nuclear marker, blue) and MyoVIIa (green) which is a HC marker. Dashed lines in (c) show the separation between the HC nuclei layer and SC nuclei layer. Arrows in (d) and (e) mark IHCs, OHCs and Hensen cells. Images in (c') and (e') show that the HCs nuclei and Hensen cells nuclei are located at the same plane. Arrows in (e') mark points of direct contact between HCs nuclei and Hensen cells nuclei. Scale bars: $10\mu m$.

Model tracking analysis:

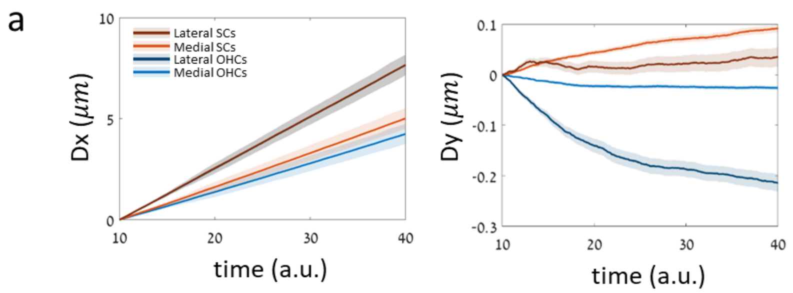

Alternative model:

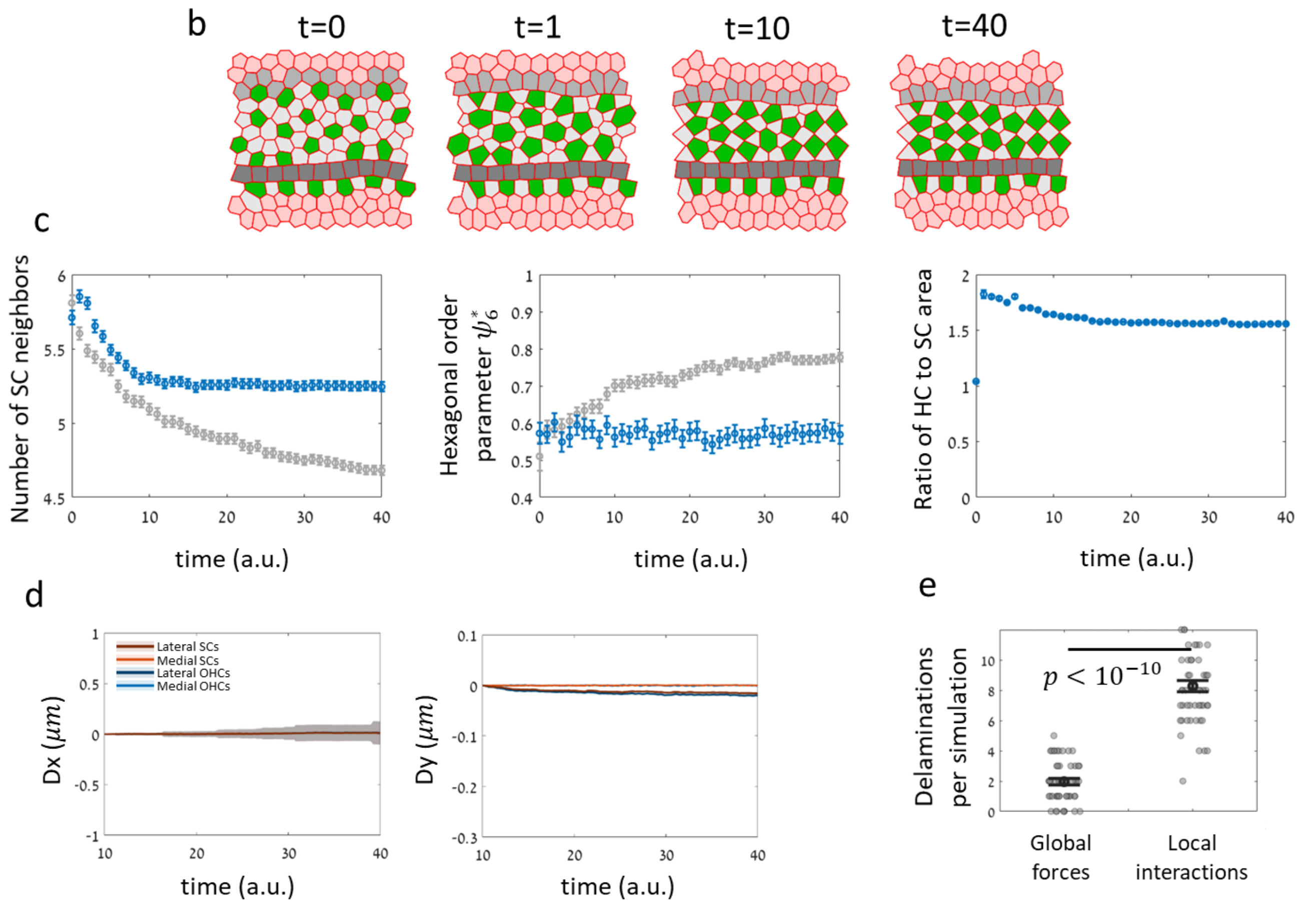

Figure S5. Model cell tracking analysis and alternative models. (a) Displacement of HCs and SCs in the model. Displacements are calculated relative to the initial position of each cell. The model captures the shear profile and separation in lateral movement observed experimentally. The data was averaged over n=50 simulations. Shaded region around every line represents the boundaries of S.E.M. **(b)** A filmstrip of a simulation from an alternative model that do not include global shear and local repulsion. The alternative model consists only of the second stage in the original model, meaning higher tension in SC:SC junctions relative to HC:SC junctions. Movie shown in Supplementary Video 10. **(c)** Simulations of the alternative model failed to capture the transition to organized pattern compared to the original model. Although the number of SC neighbors decreased, the HC organization did not exhibit higher hexagonal order (blue markers), and simulations from the original model (grey markers) produced order parameters closer to those observed experimentally. The data was averaged over n=50

simulations. Error bars indicate S.E.M. Full description of the simulations is provided in the methods. Parameters used are provided in Supplementary Table 1. **(d)** Displacement of HCs and SCs in the alternative model. Displacements are calculated relative to the initial position of each cell. The alternative model fails to capture the shear profile and separation in lateral movement observed experimentally. The data was averaged over n=50 simulations. Shaded region around every line represents the boundaries of S.E.M. **(e)** Average number of delaminations per simulation. Gray dots correspond to individual data points obtained from n=50 simulations. Black dots and error bars represent average and S.E.M, respectively This data shows that the alternative model has significantly higher number of delaminations compared to the original model. Statistical analysis was done using two-sided two-sample t-test.

Parameter sensitivity analysis:

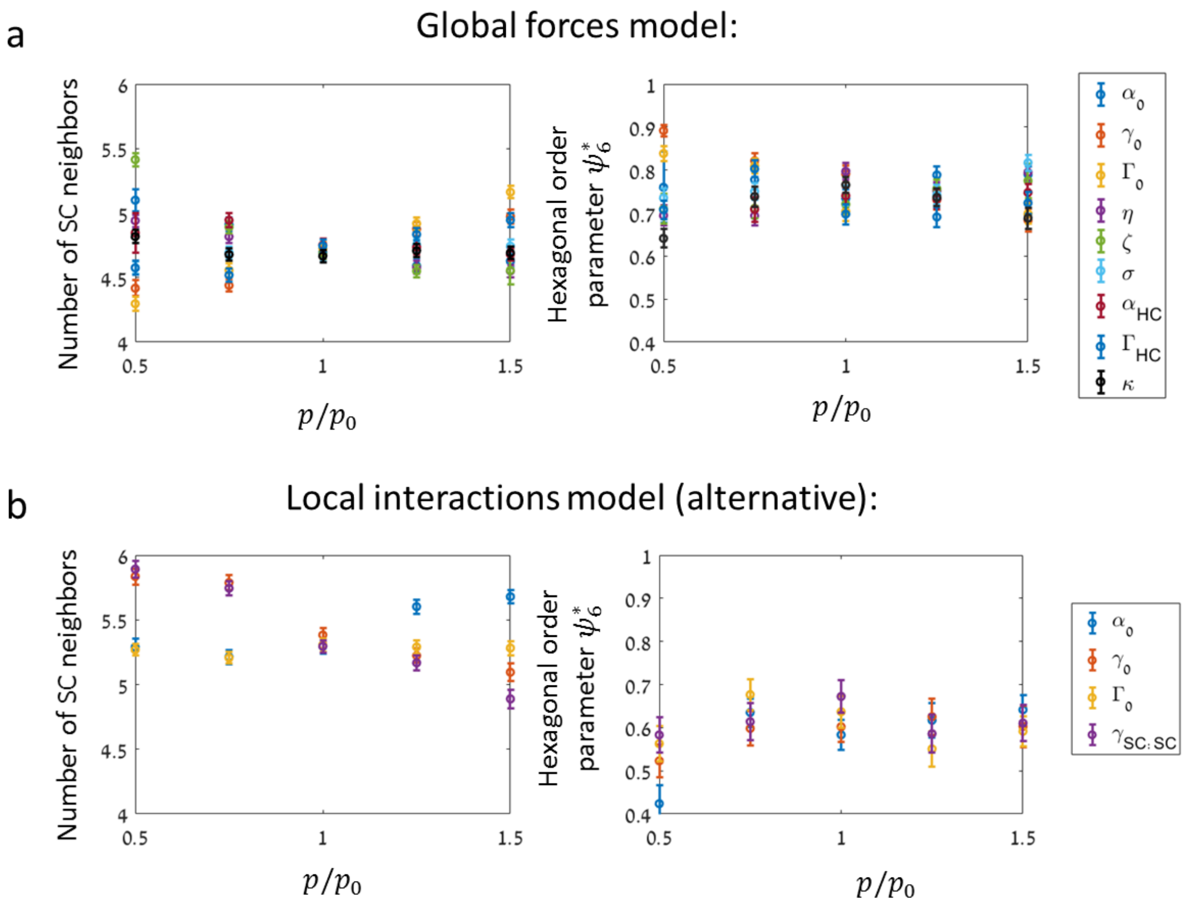

Figure S6. Model parameters sensitivity analysis. (a-b) Parameters sensitivity of the original model (a) and alternative model (b). Order parameters produced by running simulations with different values of model parameters, changing one parameter at a time. In the original model, the parameters were evaluated at the end of the 1st stage of the simulation. p/p_0 represents a normalized value of the model parameter, where p_0 is the value of the parameter as shown in Supplementary Table 1. All other parameters remain at their original values (Supplementary Table 1). Legend indicate parameters used in the analysis. This data shows that changes of ±50% in the model parameters produced relatively small changes in the values of order parameters, showing that the model does not require fine tuning of the parameters.

SCs protrusions shift

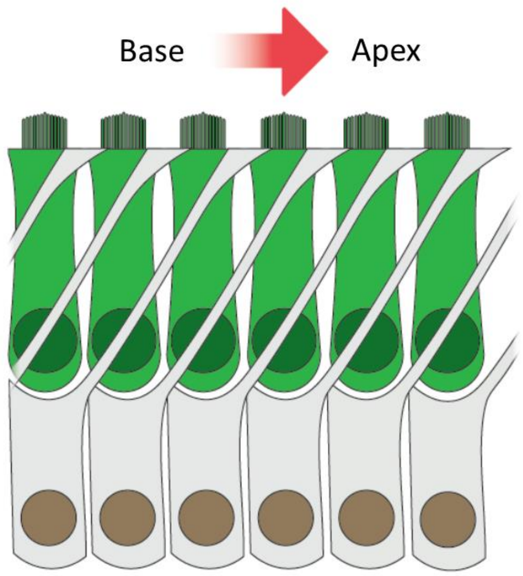

Figure S7. SCs protrusions shift. Schematic of a cross section of the organ of Corti perpendicular to the medial-lateral direction. The figure demonstrates the shift of the apical protrusions of the SCs towards the apex, observed in fully developed cochleae.

Simulation	Parameters
Figure 3c (stage 1)	$\alpha_0 = 12$, $A_0 = \frac{4\pi^2}{N_c}$, $\gamma_0 = 0.3$, $\Gamma_0 = 0.15$, $\eta = 0.5$, $\zeta = 1$, $\sigma = 1$, $\kappa = 12$, $D = 0.7$, $\alpha_{HC} = 2\alpha_0$, $\Gamma_{HC} = 2\Gamma_0$, $\alpha_{not-HC} = \alpha_0$, $\Gamma_{not-HC} = \Gamma_0$, $\gamma_{SC:SC} = \gamma_0$, $\gamma_{HC:HC} = 5\gamma_0$, $\gamma_{HC:SC}$=γ_0, $\gamma_{PC:HC} = 10\gamma_0$, $\gamma_{PC:SC} = 10\gamma_0$, $\gamma_{PC:General} = 10\gamma_0$, $\gamma_{Top:SC} = 10\gamma_0$, $\gamma_{Top:HC} = 10\gamma_0$, $\gamma_{else} = \gamma_0$
Figure 3e (stage 2)	$\alpha_0 = 12$, $A_0 = \frac{4\pi^2}{N_c}$, $\gamma_0 = 0.3$, $\Gamma_0 = 0.15$, $\eta = 0.5$, $\zeta = 1$, $\sigma = 1$, $\kappa = 12$, $D = 0.7$, $\alpha_{HC} = 2\alpha_0$, $\Gamma_{HC} = 2\Gamma_0$, $\alpha_{not-HC} = \alpha_0$, $\Gamma_{not-HC} = \Gamma_0$, $\gamma_{SC:SC} = 3\gamma_0$, $\gamma_{HC:HC} = 5\gamma_0$, $\gamma_{HC:SC}$=γ_0, $\gamma_{PC:HC} = 10\gamma_0$, $\gamma_{PC:SC} = 10\gamma_0$, $\gamma_{PC:General} = 10\gamma_0$, $\gamma_{Top:SC} = 10\gamma_0$, $\gamma_{Top:HC} = 10\gamma_0$, $\gamma_{else} = \gamma_0$
Figure S4a (alternative)	$\alpha_0 = 12$, $A_0 = \frac{4\pi^2}{N_c}$, $\gamma_0 = 0.3$, $\Gamma_0 = 0.15$, $\eta = 0$, $\zeta = 0$, $\sigma = 0$, $\kappa = 12$, $D = 0.7$, $\alpha_{HC} = \alpha_0$, $\Gamma_{HC} = \Gamma_0$, $\alpha_{not-HC} = \alpha_0$, $\Gamma_{not-HC} = \Gamma_0$, $\gamma_{SC:SC} = 3\gamma_0$, $\gamma_{HC:HC} = 5\gamma_0$, $\gamma_{HC:SC}$=γ_0, $\gamma_{PC:HC} = 10\gamma_0$, $\gamma_{PC:SC} = 10\gamma_0$, $\gamma_{PC:General} = 10\gamma_0$, $\gamma_{Top:SC} = 10\gamma_0$, $\gamma_{Top:HC} = 10\gamma_0$, $\gamma_{else} = \gamma_0$
Figure 4a (blebbistatin)	$\alpha_0 = 12$, $A_0 = \frac{4\pi^2}{N_c}$, $\gamma_0 = 0.1$, $\Gamma_0 = 0.15$, $\eta = 0$, $\zeta = 0$, $\sigma = 1$, $\kappa = 12$, $D = 0.7$, $\alpha_{HC} = 2\alpha_0$, $\Gamma_{HC} = 2\Gamma_0$, $\alpha_{not-HC} = \alpha_0$, $\Gamma_{not-HC} = \Gamma_0$, $\gamma_{all} = \gamma_0$

Supplementary Table 1. Model parameters used in simulations.

Name	Forward	Reverse
**GFP**	TCCTTGAAGAAGATGGTGCG	AAGTTCATCTGCACCACCG

Supplementary Table 2. List of primers.

Appendix – calculation of center of mass derivatives

Although all the variables in eq. 1-2 are a function of the vertices position, the center of mass of a cell does not have a trivial form. In order to calculate the center of mass of the cells in the model, we use the formula for the center of mass of a n-sided polygon. Given an ordered set of vertices position $\{x_i, y_i\}$ with $i = 0,1 \dots n-1$, the center of mass coordinated are:

$$x_{cm} = \frac{1}{6A}\sum_{i=0}^{n-1}(x_i + x_{i+1})(x_i y_{i+1} - x_{i+1} y_i)$$

$$y_{cm} = \frac{1}{6A}\sum_{i=0}^{n-1}(y_i + y_{i+1})(x_i y_{i+1} - x_{i+1} y_i)$$

where the area of the polygon A is given by:

$$A = \frac{1}{2}\sum_{i=0}^{n-1}(x_i y_{i+1} - x_{i+1} y_i)$$

In these formulas we define $x_n \equiv x_0$ and $y_n \equiv y_0$. As part of the derivatives in eq. 3, we use the derivatives of the center of mass relative to the positions of the vertices. For one of the vertices, x_k, y_k these derivatives are given by:

$$\frac{\partial x_{cm}}{\partial x_k} = -\frac{1}{A}\frac{\partial A}{\partial x_k}x_{cm} + \frac{1}{6A}\left[2x_k y_{k+1} - 2x_k y_{k-1} - x_{k+1} y_k + x_{k-1} y_k + x_{k+1} y_{k+1} - x_{k-1} y_{k-1}\right]$$

$$\frac{\partial y_{cm}}{\partial x_k} = -\frac{1}{A}\frac{\partial A}{\partial x_k}y_{cm} + \frac{1}{6A}\left[(y_k + y_{k+1})y_{k+1} - (y_{k-1} + y_k)y_{k-1}\right]$$

$$\frac{\partial A}{\partial x_k} = \frac{1}{2}(y_{k+1} - y_{k-1})$$

$$\frac{\partial x_{cm}}{\partial y_k} = -\frac{1}{A}\frac{\partial A}{\partial y_k}x_{cm} + \frac{1}{6A}\left[(x_{k-1} + x_k)x_{k-1} - (x_k + x_{k+1})x_{k+1}\right]$$

$$\frac{\partial y_{cm}}{\partial y_k} = -\frac{1}{A}\frac{\partial A}{\partial y_k}y_{cm} + \frac{1}{6A}\left[2y_k x_{k-1} - 2y_k x_{k+1} + x_k y_{k+1} - x_k y_{k-1} + y_{k-1} x_{k-1} - y_{k+1} x_{k+1}\right]$$

$$\frac{\partial A}{\partial y_k} = \frac{1}{2}(x_{k-1} - x_{k+1})$$

In the repulsive term in eq. 1, the center of mass is hidden in R_{nm}:

$$E_{nm}^{rep} = \sigma\left(\frac{D}{R_{nm}}\right)^{\kappa}, \quad \vec{R}_{nm} = \vec{R}_n^{CM} - \vec{R}_m^{CM}, \quad R_{nm} = \left|\vec{R}_{nm}\right| = \sqrt{(x_n^{CM} - x_m^{CM})^2 + (y_n^{CM} - y_m^{CM})^2}$$

where $\vec{R}_n^{CM}, \vec{R}_m^{CM}$ are the center of mass position of the cells. For a vertex k of cell n, the derivative of this energy term with respect to the position of vertex k is calculated as:

$$\frac{\partial E_{nm}^{rep}}{\partial x_k} = \frac{\partial E_{nm}^{rep}}{\partial R_{nm}}\frac{\partial R_{nm}}{\partial x_k} = \frac{\partial E_{nm}^{rep}}{\partial R_{nm}}\left(\frac{\partial R_{nm}}{\partial x_n^{CM}}\frac{\partial x_n^{CM}}{\partial x_k} + \frac{\partial R_{nm}}{\partial y_n^{CM}}\frac{\partial y_n^{CM}}{\partial x_k}\right)$$

$$\frac{\partial E_{nm}^{rep}}{\partial y_k} = \frac{\partial E_{nm}^{rep}}{\partial R_{nm}}\frac{\partial R_{nm}}{\partial y_k} = \frac{\partial E_{nm}^{rep}}{\partial R_{nm}}\left(\frac{\partial R_{nm}}{\partial x_n^{CM}}\frac{\partial x_n^{CM}}{\partial y_k} + \frac{\partial R_{nm}}{\partial y_n^{CM}}\frac{\partial y_n^{CM}}{\partial y_k}\right)$$
